# Protective effects on liver, kidney and pancreas of enzymatic- and acidic-hydrolysis of polysaccharides by spent mushroom compost (*Hypsizigus marmoreus*)

**DOI:** 10.1038/srep43212

**Published:** 2017-02-24

**Authors:** Min Liu, Xinling Song, Jianjun Zhang, Chen Zhang, Zheng Gao, Shangshang Li, Huijuan Jing, Zhenzhen Ren, Shouxian Wang, Le Jia

**Affiliations:** 1 College of Life Science, Shandong Agricultural University, Taian, 271018, PR China; 2 Institute of Plant and Environment Protection, Beijing Academy of Agriculture and Forestry Sciences, Beijing Engineering Research Center for Edible Mushroom, Key Laboratory of Urban Agriculture (North), Ministry of Agriculture, Beijing, PR China

## Abstract

The present work investigated the protective effects on liver, kidneys and pancreas of spent mushroom compost polysaccharide (SCP) and its hydrolysates (enzymatic- (ESCP) and acid-hydrolyzed SCP (ASCP)) from *Hypsizigus marmoreus*, in streptozotocin (STZ)-induced diabetic mice. The results showed that enzymatic (superoxide dismutase (SOD), glutathione peroxidase (GSH-Px) and catalase (CAT)) and non-enzymatic activities (total antioxidant capacity (T-AOC)) were significantly increased, the lipid peroxide contents (lipid peroxide (LPO) and malonaldehyde (MDA)) were remarkably reduced, and the clinical parameters were observably mitigated in diabetic mice treated with these three polysaccharides. Furthermore, histological observations also indicated recovery. These conclusions demonstrated that both SCP and its hydrolysates ESCP and ASCP possessed potent antioxidant activities and can be used as a potentially functional food for the prevention of diabetes and its complications induced by STZ.

Diabetes mellitus (DM), an endocrine metabolic disease that is clinically characterized by chronic hyperglycemia due to deficient insulin action, is considered a major health risk worldwide^1^,^2^. DM can induce pathological damage to the liver, kidneys and pancreas, with characteristic abnormalities in the metabolism of carbohydrates, lipids and proteins^3^,^4^. Epidemiological studies have suggested that the incidence of DM is influenced by many aspects, including genetic predisposition, diet and environmental elements^5^. Recent literature has demonstrated that oxidative stress, reflected by the overproduction of reactive oxygen species (ROS) and an inferior antioxidant defense, is a participant in accelerating the progress of DM and its complications^6^,^7^. Hence, dietary or clinical oxidant supplements could be beneficial in protecting against DM^3^. However, many reports have testified that synthetic antioxidant agents are toxic and can induce serious side effects in clinical practice^8^. Therefore, natural substances with superior antioxidant activities in inhibiting oxidative-stress-induced damage have become an attractive therapeutic strategy for reducing the risk of DM.

Artificial mushrooms, a traditional Chinese source of natural medicines and functional foods, have been widely used in the prevention and treatment of numerous diseases^9^,^10^,^11^. Mature and large-scale artificial cultivation have resulted in approximately five million tons of spent mushroom compost (SMC) annually, which generates many environmental pollution and public health issues^12^. SMC also contains residual mycelia, carbohydrates, organic substances, inorganic ions, and residual enzymes including cellulose, hemicellulose, and ligninase. Traditionally, research on SMC has focused on fundamental utilization such as biological feed and organic fertilizer^13^. Further efficient utilization of SMC is urgently necessary.

Among the various nutrient substances obtained from mushrooms, polysaccharides have been demonstrated to possess a diversity of useful biological properties including anti-oxidant, anti-tumor, anti-bacterial, anti-inflammatory, immunomodulatory, anti-hyperglycemic and anti-hypercholesterolemic activities^14^. In light of these scientific findings, many investigators have devoted themselves to assessing the anti-diabetic effects of polysaccharides from either the fruiting body or mycelia, such as *Phellinus baumii*^15^, and *Catathelasma ventricosum*^16^. Furthermore, many studies have indicated that the polysaccharides extracted from the SMC of *Flammulina velutipes*^17^, *Agrocybe cylindracea*^18^, and *Pleurotus eryngii*^19^ have potential effects in exploiting new drugs and biological compounds for intervening in human disease. *Hypsizigus marmoreus*, an essential species of industrial cultivation, has been accepted by consumers due to its medicinal properties and characteristic mouthfeel^20^. Polysaccharides extracted from either fruiting bodies or mycelia of *H. marmoreus* have received increasing academic attention and have been extensively used for the prevention of organ damage induced by chemical toxicants such as carbon tetrachloride^21^ and galactose^22^. Nevertheless, the anti-diabetic effects of SMC polysaccharides (SCP) remain poorly understood. Therefore, one water-soluble polysaccharide (SCP) and its two hydrolyzates (enzymatic- (ESCP) and acidic-hydrolysis SCP (ASCP)) were extracted and prepared for this study. The protective effects on the liver, kidneys and pancreas, and the antioxidant activities of the three polysaccharides in streptozocin (STZ)-induced diabetic mice were investigated. In addition, monosaccharide compositions were characterized.

## Results

### Monosaccharide compositions analysis

**Figure 1:**
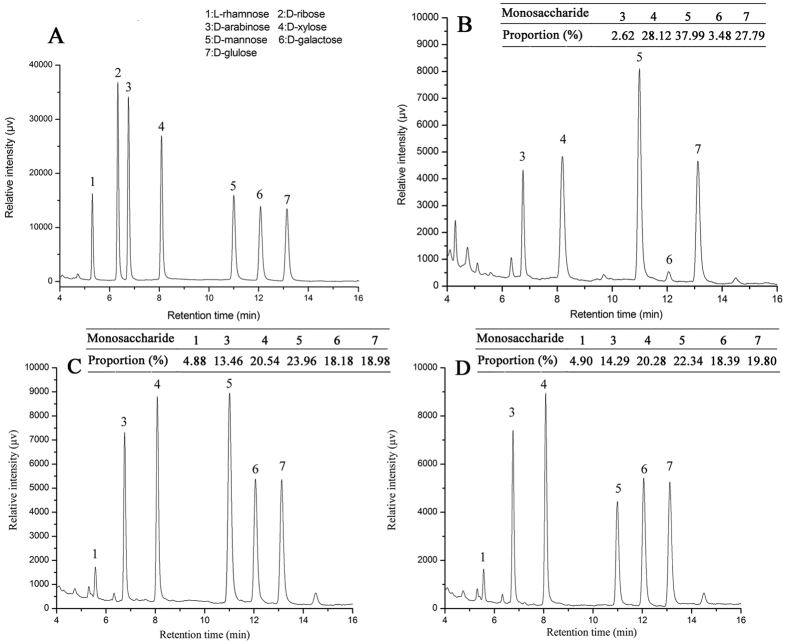
The monosaccharide compositions of (**A**) Standard, (**B**) SCP, (**C**) ESCP, and (**D**) ASCP.

The monosaccharide compositions of ESCP, ASCP and SCP were identified by comparing their retention times to those of standards (Fig. 1). As shown in Fig. 1, SCP was composed of D-arabinose, D-xylose, D-mannose, D-galactose and D-glucose in mass percentages of 2.62%, 28.12%, 37.99%, 3.48% and 27.29% with a molar ratio of 0.46:4.93:6.66:0.61:4.87 (Fig. 1B). ESCP was composed of L-rhamnose, D-arabinose, D-xylose, D-mannose, D-galactose and D-glucose in mass percentages of 4.88%, 13.46%, 20.54%, 23.96%, 18.18% and 18.98% with a molar ratio of 1.41:3.89:5.94:6.92:5.26:5.49 (Fig. 1C). ASCP contained L-rhamnose, D-arabinose, D-xylose, D-mannose, D-galactose and D-glucose in mass percentages of 4.90%, 14.29%, 20.28%, 22.34%, 18.39% and 19.80% with a molar ratio of 1.35:3.94:5.59:6.16:5.07:5.46 (Fig. 1D), respectively. These results indicated that the predominant monosaccharide in ESCP and ASCP was superior in L-rhamnose compared to SCP.

### Acute toxicity study

During the whole gavage of ESCP, ASCP and SCP, even at doses as high as 4,000 mg/kg, no mice showed any clinical signs of toxicity. Furthermore, no deaths were observed originally or at the end of the treatment, indicating that these three polysaccharides are non-toxic substances^23^.

### The influence of SCP, ESCP and ASCP on animal experiments

**Figure 2:**
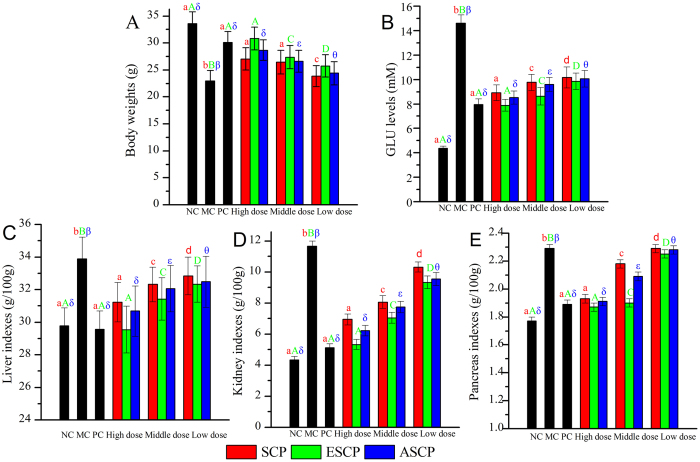
Effects of SCP, ESCP and ASCP on (**A**) Body weight, (**B**) GLU level, (**C**) Liver index, (**D**) Kidney index, (**E**) Pancreas index. The values are reported as the means ± SD (n = 5). Bars with no letters in common are significantly different (*P* < 0.05).

The effects of SCP, ESCP and ASCP on body weights, blood glucose (GLU) levels and indices of liver, kidneys and pancreas damage in STZ-induced diabetic mice are illustrated in Fig. 2.

As demonstrated in Fig. 2A, after injection of STZ, the mice in model control (MC) group showed a significant loss in body weight, in accordance with the clinical representation of emaciation. After administration of SCP, ESCP and ASCP in diabetic mice at a high dose of 800 mg/kg, body weights were increased by 17.79%, 34.39% and 24.98%, respectively, when compared with the MC group (*P* < 0.05), while body weights were 15.34%, 19.31% and 15.95% higher at a middle dose of 400 mg/kg, as well as 4.05%, 12.16% and 8.49% higher at a low dose of 200 mg/kg compared to MC group.

The diabetic mice showed a significant increase in GLU levels (Fig. 2B). However, this elevation could be prevented by treatment with SCP, ESCP and ASCP in concentration-dependent patterns at the tested concentrations, indicating that both SCP and its hydrolyzed substances had potential effects in remitting the hyperglycemic stress. After treatment with SCP, ESCP and ASCP at the highest dose (800 mg/kg), the GLU levels of mice decreased by 38.90%, 46.03% and 41.58%, respectively, when compared with diabetic mice (MC group).

The liver, kidney and pancreas indexes are shown in Fig. 2C–E. Significant increases were observed in STZ-induced diabetic mice when compared with normal control (NC) mice (*P* < 0.05). However, these pathological increases could be mitigated by treatment with three polysaccharides in a dose-dependent manner. The high dose of ESCP had the greatest potential effect on alleviating organ damage. Briefly, after the administration of ESCP at a high dose, the liver, kidney, and pancreas indexes significantly decreased by 12.84%, 38.90% and 15.72%, while these indexes significantly decreased by 7.87%, 45.48% and 17.47% after treatment with SCP and 9.47%, 46.03% and 18.34% after treatment with ASCP in comparison with MC group at the same dose, respectively.

**Figure 3:**
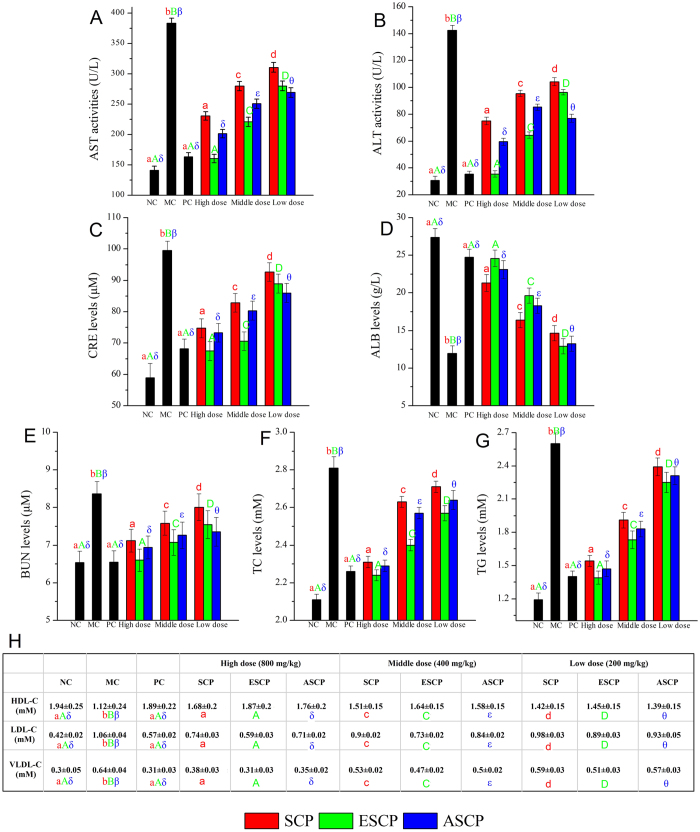
Effects of SCP, ESCP and ASCP on serum properties. (**A**) AST activities, (**B**) ALT activities, (**C**) CRE levels, (**D**) ALB levels, (**E**) BUN levels, (**F**) TC levels, (**G**) TG levels, and (**H**) HDL-C, LDL-C and VLDL-C levels. The values are reported as the means ± SD (n = 5). Bars with no letters in common are significantly different (*P* < 0.05).

As shown in Fig. 3, significant increases in aspartate aminotransferase (AST), alanine aminotransferase (ALT), creatinine (CRE), and urea nitrogen (BUN), as well as decreased albumin (ALB) were observed in STZ-induced mice when compared with NC mice. Interestingly, these increases could be notably inhibited, while decreases could be significantly enhanced by treatment with the three polysaccharides, especially in the ESCP treatment group at high dose of 800 mg/kg. The ESCP group did not significantly differ from the positive control mice treated with glibenclamide, indicating that ESCP had the greatest ability to protect the liver, kidneys and pancreas against STZ.

The levels of lipids and lipoproteins in serum, such as total cholesterol (TC), triacylglycerol (TG), high density lipoprotein cholesterol (HDL-C), low density lipoprotein cholesterol (LDL-C) and very low density lipoprotein cholesterol (VLDL-C), are depicted in Fig. 3F–H. The TC, TG, LDL-C and VLDL-C levels in diabetic mice notably increased (*P* < 0.05), while the HDL-C level was remarkably decreased compared with NC mice (*P* < 0.05). After administration of glibenclamide and three different polysaccharides, the lipid and lipoprotein levels in diabetic mice recovered significantly (*P* < 0.05), indicating that polysaccharides extracted from the SMC of *H. marmoreus* had potential effects on improving lipid metabolism.

**Figure 4:**
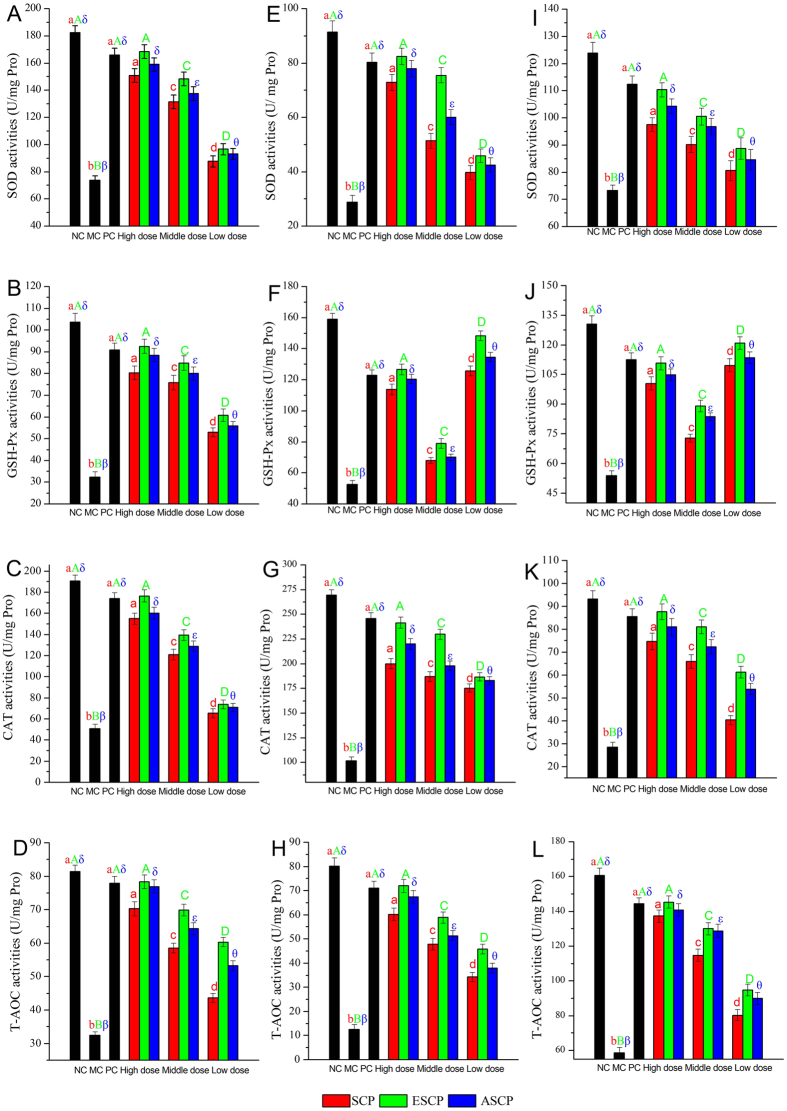
Effects of SCP, ESCP and ASCP on the activities of SOD, GSH-Px, CAT, T-AOC in hepatic homogenates (**A–D**), renal homogenates (**E–H**), and pancreatic homogenates (**I–L**), respectively. The values are reported as the means ± SD (n = 5). Bars with no letters in common are significantly different (*P* < 0.05).

As Fig. 4 shows, significant reductions in superoxide dismutase (SOD), glutathione peroxidase (GSH-Px), catalase (CAT) and total antioxidant capacity (T-AOC) activities were observed in STZ-induced diabetic mice compared to the NC group (*P* < 0.05), indicating that the diabetic mice endured serious oxidative stress. However, these reductions in parameters reflecting oxidative stress could be significantly enhanced by treatment with SCP, ESCP, and ASCP at three doses (*P* < 0.05). The hepatic SOD, GSH-Px, CAT and T-AOC activities reached the maximum in ESCP-treated mice at high doses, which were 128.29%, 185.49%, 137.34% and 141.21% higher than in MC group, respectively. Hepatic activities were 115.40%, 173.30%, 116.25% and 136.93% higher in mice treated by ASCP and 104.58%, 147.98%, 96.60% and 116.51% higher in mice treated by SCP than those in MC group at the same dosage. A similar tendency of ESCP on SOD, CAT, and T-AOC activities was observed in the kidneys and pancreas. The renal and pancreatic SOD, CAT and T-AOC activities in ESCP treatment groups at a dose of 800 mg/kg were 186.38%, 247.86% and 147.39%, as well as 50.66%, 207.44% and 472.32% higher than those in MC group. These were all relatively higher than in mice treated with ASCP and SCP at the same concentrations, respectively. However, the GSH-Px activities of polysaccharides in the kidneys and pancreas were not dose-dependent (Fig. 4F and J). Glibenclamide, as a positive control in PC groups, also effectively protected organs against decreased SOD, GSH-Px, CAT and T-AOC activities (*P* < 0.05).

**Figure 5:**
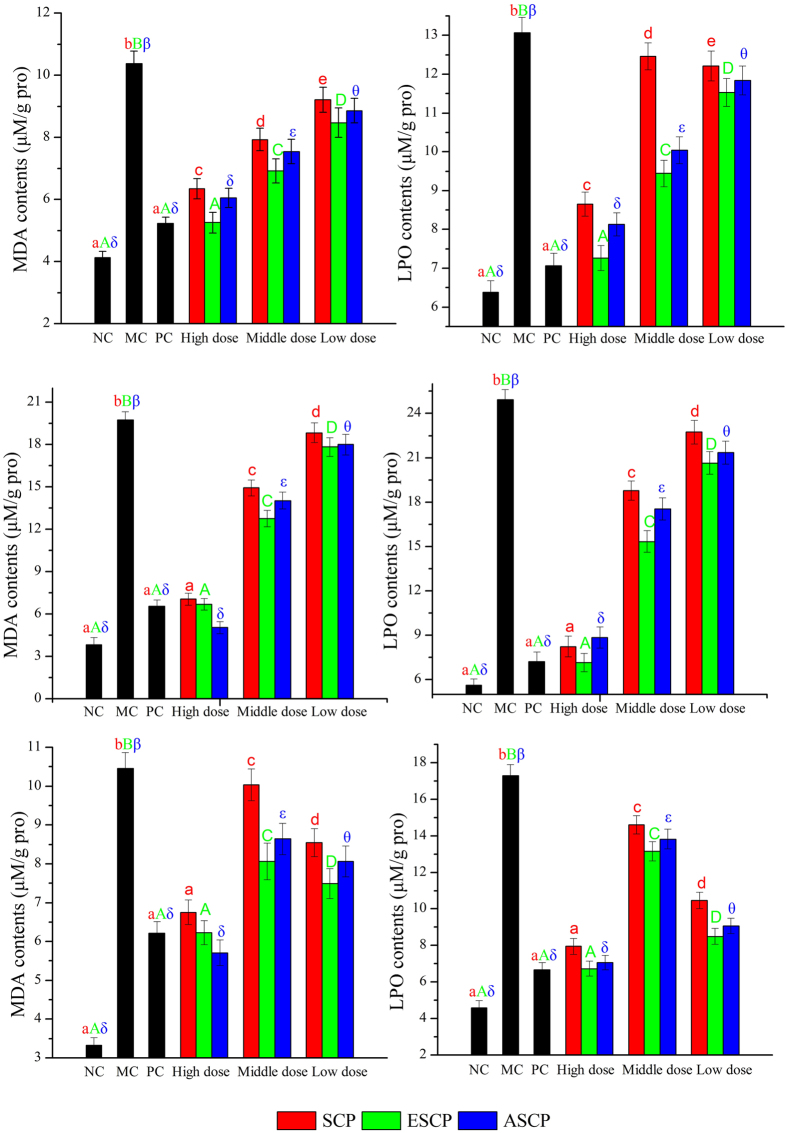
Effects of SCP, ESCP and ASCP on levels of MDA (**A**), LPO (**B**) in hepatic homogenates, MDA (**C**), LPO (**D**) in renal homogenates, and MDA (**E**), LPO (**F**) in pancreatic homogenates, respectively. The values are reported as the means ± SD (n = 5). Bars with no letters in common are significantly different (*P* < 0.05).

In this study, lipid peroxidation, including lipid peroxide (LPO) and malonaldehyde (MDA), was also examined. As shown in Fig. 5, both the MDA and LPO contents in the tissue homogenates of diabetic mice increased significantly in comparison with mice in the NC group (no dose dependence). After treatment with the three polysaccharides, these tendencies were inhibited, suggesting that at high doses, the three polysaccharides had potential protective effects. In ESCP treated mice at 800 mg/kg, the hepatic MDA and LPO content decreased by 49.37% and 44.41%, while the renal MDA and LPO content decreased by 66.21% and 71.35%, and the pancreatic MDA and LPO contents decreased by 40.44% and 61.05%. Similar conclusions could be drawn from treatment with ASCP and SCP, demonstrating that ESCP, ASCP, and SCP had inhibitory effects on lipid peroxidation. Glibenclamide-treated mice also manifested significant declines in MDA and LPO contents compared to MC mice (PC groups, *P* < 0.05).

### Histopathological observations

**Figure 6:**
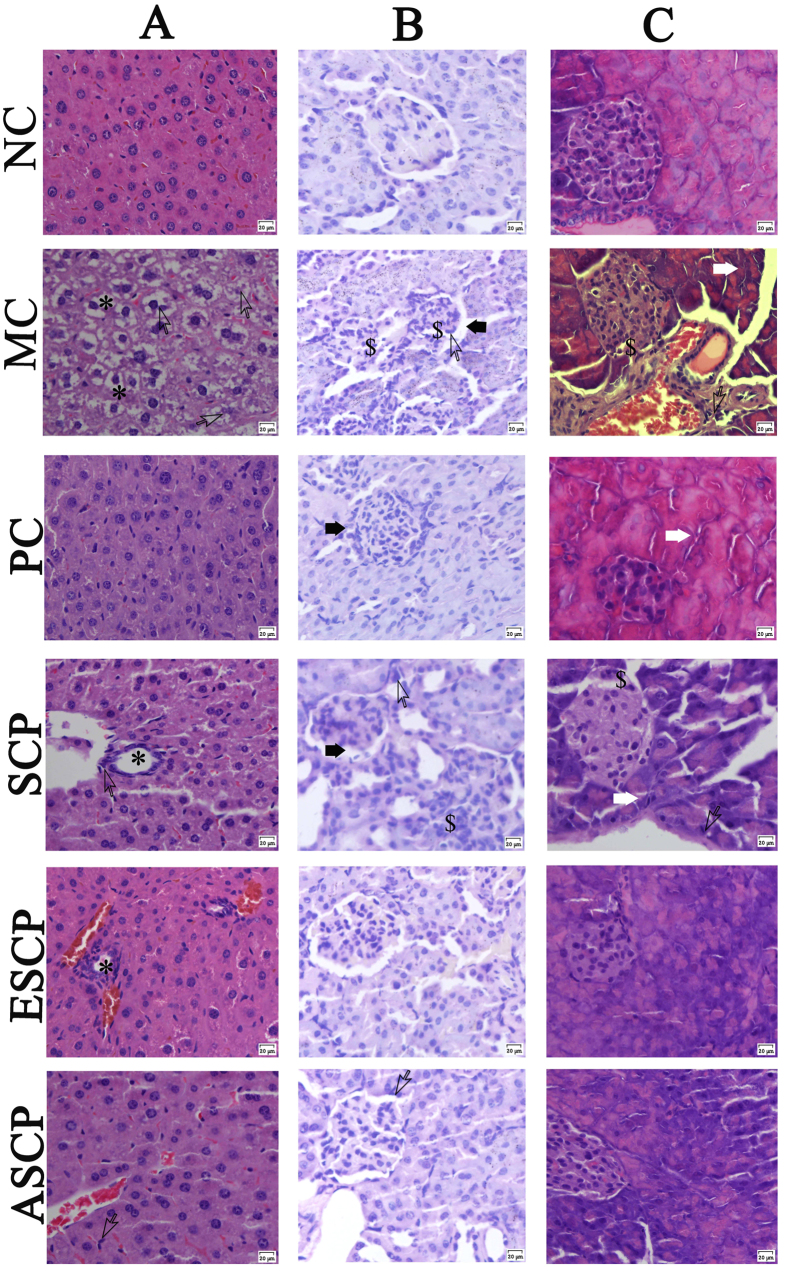
Effects of SCP, ESCP and ASCP treatment on tissues damage in STZ-induced diabetic mice at the high dose (800 mg/kg) (Hematoxylin/eosin staining, magnification 400X). (**A**) Liver, (**B**) Kidney, (**C**) Pancreas. Cellular degeneration and nucleus degradation (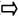 open arrowheads), lipid droplet accumulation (*), glomerulus destruction, tubulointerstitial lesions, glomerular sclerosis and atrophy of the islets ($), vacuolation of tubular epithelial cells and loss of brush border (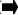 black arrowheads), and congestion of the central vein (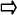 white arrowheads).

Photomicrographs of the histochemical staining of the liver, kidney cortex, and pancreas were shown in Fig. 6. Obviously, the organs damage was mainly evidenced by the cellular and nucleus degradations. Besides, the diabetic mice showed other morphologies in these organs, such as lipid droplet accumulation in liver (Fig. 6A), glomerulus destruction, tubulointerstitial lesions, glomerular sclerosis, vacuolation of tubular epithelial cells, and loss of brush border in kidney (Fig. 6B), as well as atrophy of the islets and congestion of the central vein in pancreas (Fig. 6C). Interestingly, the severe hepatic, renal and pancreatic lesions induced by STZ were considerably prevented by administration of ESCP, ASCP and SCP at high doses (800 mg/kg, Fig. 6). The morphologies of these three tissues were similar to these of mice in NC groups, indicating that ESCP, ASCP and SCP can protect these tissues from acute STZ-intoxication. The histopathologies of ESCP, ASCP and SCP at low (200 mg/kg) and middle (400 mg/kg) doses are shown in Supplementary File 1 (Figure S1).

## Discussion

In the present study, we characterized the monosaccharide compositions of SCP and its two hydrolysates, ASCP and ESCP, and demonstrated for the first time that the oral administration of polysaccharides could effectively alleviate hyperglycemia, dyslipidemia, oxidative stress and organ injury in STZ-induced diabetic mice. These achievements suggest that SCP, ASCP, and ESCP are potential bioactive compounds responsible for anti-diabetic effects and thus can provide new insight into the potential mechanisms of anti-diabetic effects of polysaccharides from the SMC of *H. marmoreus* and cyclic utilizations of SMC.

Previous studies have demonstrated that STZ, a toxic agent produced by *Streptomyces achromogenes*, can induce apoptosis of organs and suppress insulin biosynthesis by stimulating the over-production of ROS and causing oxidative damage^24^,^25^. The diabetogenic effects of STZ became more susceptible to the development of significant hyperglycemia and its complications. In the present work, Kunming mice were intraperitoneally injected with a low dose of STZ (80 mg/kg) to induce diabetes. A significant loss in body weight and a sharp increase in GLU levels were observed in STZ-induced diabetic mice, and the mice showed serious clinical symptoms, including swelling of tissues as reflected by the organs’ indexes over a period of 15 days. Meanwhile, diabetic mice in MC group exhibited continuous signs of diabetes. Many literature reports have demonstrated that decreased body weight and increased GLU levels can be ascribed to unavailability of carbohydrate metabolism^26^,^27^. Interestingly, these morbid variations could be alleviated by treatment with SCP, ASCP and ESCP, indicating that these three polysaccharides have potential effects in improving glucose homeostasis.

During the impairment of carbohydrate metabolism, the liver, kidneys and pancreas also play vital roles in the glucose metabolism of diabetic mice^28^. Clinically, several enzymes and substance levels are used as biochemical markers for early diagnosis of diabetes and its complications, including AST, ALT, BUN, CRE and ALB. In the present work, mice showed liver damage as reflected by markedly elevated enzymatic activities of serum AST and ALT, indicating that diabetes can cause liver damage. Serum analysis of ALB, BUN and CRE levels has been used to reflect the physical status of the kidney^29^. From our work, the administration of ESCP at high doses (800 mg/kg) had significant effects on the suppression of AST and ALT activities as well as BUN, CRE, and ALB levels in serum, suggesting that ESCP had potential protective effects against STZ-toxicity to the liver, kidneys and pancreas.

Furthermore, dysregulation of lipid metabolism, which is characterized by elevated levels of TG, TC, LDL-C, and VLDL-C and decreased levels of HDL-C, is a vital determinant of the course and status of DM and its complications. These changes also impose increased risk of coronary heart disease^30^. Many studies have demonstrated that HDL-C, which can act as an antioxidant and promote the efflux of TC and TG from peripheral tissues to the liver for catabolism during circulation, is useful for human health. In contrast, excess LDL-C and VLDL-C levels can deposit in blood vessel walls, leading to the formation of atherosclerotic plaque lesions^31^,^32^. Therefore, low levels of HDL-C and high levels of LDL-C and VLDL-C were dangerous for mice^33^. In the present work, administration of polysaccharides significantly lowered TC, TG, LDL-C and VLDL-C levels and remarkably elevated HDL-C levels in diabetic mice, suggesting that both SCP and its hydrolysates (ESCP and ASCP) had protective effects on the liver, kidneys and pancreas, due to the low risk of cardiovascular disease and positive effects on lipid profiles.

The literature suggests that organ damage is usually caused by oxidative stress, defined as a persistent imbalance between the production of ROS and antioxidant defenses. Hence, enhanced oxidative stress was proposed as a vital contributor to the progress of DM and its complications^34^. Commonly, SOD first reduces superoxide to hydrogen peroxide, and GSH-Px and CAT catalyze hydrogen peroxide to water^35^,^36^,^37^. MDA and LPO, produced by the interaction between ROS and polyunsaturated fatty acids, are frequently used as indicators of oxidative stress. GSH-Px can also catalyze lipid hydroperoxides to their corresponding alcohols, preventing the formation of MDA and LPO^37^,^38^,^39^. In this study, the significant increase in MDA and LPO levels and decrease in SOD, GSH-Px and CAT activities found in the liver, kidneys and pancreas of STZ-induced diabetic mice illustrated the enhanced oxidative stress in these organs. However, enzyme activities were increased and lipid peroxidation decreased after treatment of the three polysaccharides, indicating that both SCP and its hydrolysates (ASCP and ESCP) had potential antioxidant effects against ROS. Corroborating these results, numerous studies have shown anti-diabetic ability by preventing a decrease in antioxidant enzyme activities and suppressing increased lipid peroxidations, demonstrating that the hypoglycemic effects of polysaccharides from the SMC of *H. marmoreus* may be due their effect on alleviation of oxidative stress^15^,^16^.

Moreover, STZ-induced diabetic mice showed serious degeneration in almost all tissues, especially in the liver, kidneys and pancreas, hepatic necrosis, glomerular sclerosis, congestion in the central vein and atrophy of islets were observed (Fig. 6). Herein, the histological observation was commonly used to obtain visual evidence on the protective capacity of the tested polysaccharides. The histopathological evaluations verified that the repair capacity of these three polysaccharides may involve the stimulation of insulin release or reduction of insulin metabolism, which were further confirmed by Das *et al*.^40^.

Additionally, it had been reported that monosaccharide compositions were attributed to the biological effects of polysaccharides^41^, and Wu *et al*.^42^ had demonstrated that polysaccharides showed superior physicochemical properties of good water solubility, high stability, safety and non-toxicity after enzymatic hydrolysis. Here, the two hydrolysates contained L-rhamnose, which indicates that this monosaccharide may play an important role in anti-diabetic and antioxidant capacities. The results were in agreement with those of Zhang *et al*.^43^ and Jia *et al*.^44^ Furthermore, the monosaccharide compositions of the three polysaccharides (Fig. 1) indicated that D-galactose could have positive effects on polysaccharide biological activities.

## Conclusions

The present investigation indicated that polysaccharides extracted from the SMC of *H. marmoreus*, particularly the ESCP, displayed effective antioxidant and hypolipidemic activities and protected the liver, kidneys and pancreas of STZ-induced diabetic mice, demonstrating that both SCP and its hydrolysates (ESCP and ASCP) have potential as functional food ingredients in the treatment of diabetes and its complications. Furthermore, the current results also provide a strategy to use SMC as a value-added material for environment protection and resource utilization.

## Materials and Methods

### Materials and chemicals

The SMC of *H. marmoreus* was provided by Shandong Ronfun Mushroom Co., Ltd. (Dongying, China). The STZ and standard monosaccharide samples were purchased from Sigma Chemicals Co., Ltd. (St. Louis, USA). The diagnostic kits for antioxidant indicators were purchased from Nanjing Jiancheng Bioengineering Ins. (Nanjing, China). All other chemicals used in the present work were purchased from the Beijing Solarbio Science & Technology Co., Ltd. (Beijing, China).

### Preparation of SCP, ESCP and ASCP

The SMC of *H. marmoreus* was dried and crushed into powder using a disintegrator (Shanghai, China). The powder was triply mixed with distilled water and treated with an ultrasonic processor (800 W, 600 s, Scientz-II D, Ningbo Scientz Biotechnology Co., Ltd., Ningbo, China). The post-treatment mixture was subjected to extraction in a thermal water bath (75 °C) for 2 h and the homogenate was centrifuged at 3,000 rpm for 10 min. The supernatant was collected and mixed with three volumes of ethanol (85%, v/v) overnight (−4 °C). The resulting precipitate, separated by centrifugation at 3,000 rpm for 15 min, was considered the SCP. ESCP and ASCP were separately prepared according to methods reported by Yang *et al*.^45^ and Huang *et al*.^46^, respectively, with slight modifications.

The ESCP was obtained through enzymatic hydrolysis with snailase (1% in sodium acetate buffer) in a ratio of substrate to enzyme of 1:4, w/v at an extraction temperature of 40 °C, pH 6, and an extraction time of 5 h. ASCP was simultaneously produced by acid hydrolysis with H_2_SO_4_ (1 M, 1:20, w/v) in boiling water for 8 h. After deproteinization^47^ and dialysis, both SCP and its hydrolysates (ESCP and ASCP) were washed with ultrapure water and lyophilized. The carbohydrate content was determined using the phenol-sulfuric acid colorimetric method with glucose as a standard^48^. SCP, ESCP, and ASCP were neutralized before further analyses.

### Monosaccharide composition analysis

Monosaccharide composition was determined using gas chromatography (GC-2010, Shimadzu, Japan) equipped with an Rtx-1 capillary column (30 mm × 0.25 mm × 0.25 μm) as previously described^49^. Composition identification was performed by comparison with standard monosaccharides (D-mannose, L-rhamnose, D-glucose, D-galactose, D-arabinose, D-ribose, and D-xylose). The relative molar ratios were calculated using the area normalization method according to the chromatogram.

### Acute toxicity study

The acute toxicity study was performed using the method reported by Chao *et al*.^50^. Twenty Kunming strain mice were randomly divided into four groups of five animals each. In the control group, mice were given free access to food and water. In the experimental group, mice were given the three polysaccharide samples per os at a dose of 4,000 mg/kg. The animals were observed continuously for any mortality and gross behavioral changes including irritation, restlessness, respiratory distress, abnormal locomotion, catalepsy and toxic symptoms.

### Animal experiments

Kunming strain mice (male, 20 ± 2 g) were purchased from Taibang Biological Products Co., Ltd. (Taiwan, China) and housed in the animal room under controlled conditions (20–25 °C, 12 h/12 h light/dark cycle) for 7 days of accommodation, during which time the mice had *ad libitum* access to food and water. The experiments were performed as approved by the Institutional Animal Care and Use Committee of Shandong Agricultural University, and in accordance with the Animals (Scientific Procedures) Act 1986 (amended 2013).

After this accommodation, the diabetic models were induced by a triple-successive (24 h time interval) intraperitoneal injection with STZ (80 mg/kg, freshly prepared in citrate buffer solution, 0.1 M, pH 4.5), with citrate buffer alone in the NC group. After a 12-h fast, all STZ-injected mice were assessed by measuring GLU levels caudally, and the diabetic models were considered successful if the GLU levels were above 13.3 mM^51^. The diabetic mice were randomly allocated into eleven groups of five mice each, including the MC group, which received distilled water only; the PC group, which received glibenclamide (20 mg/kg); and nine dose experiment groups treated with SCP, ESCP and ASCP at high- (800 mg/kg), middle- (400 mg/kg) and low-level (200 mg/kg) doses^52^. Mice in the NC group received distilled water alone. The gavages were processed with a syringe daily and continued for 15 successive days. The body weight of mice was monitored daily. At the end of the experiment, all mice were fasted overnight and were then sacrificed by exsanguinations under diethyl ether anesthesia.

Blood samples from the orbital sinus were centrifuged at 14,000 rpm (4 °C, 10 min) to afford the required serums. ALT and AST, as well as GLU, ALB, BUN, CRE, HDL-C, LDL-C, VLDL-C, TC and TG were measured using an automatic biochemical analyzer (ACE, USA).

The livers, kidneys and pancreases were rapidly removed, weighed and immediately homogenized (1:9, w/v) in phosphate buffer (0.2 M, pH 7.4, 4 °C). After centrifugation (5,000 rpm, 4 °C) for 20 min, the supernatants were collected for further biochemical analysis. The organ index was calculated according to the following formula: organ weight/body weight × 100. The activities of SOD, GSH-Px, CAT and T-AOC, as well as the LPO and MDA content, were determined using commercial reagent kits according to the instructions.

Organ samples were fixed in a 4% paraformaldehyde solution and embedded in paraffin. The slices (7-μm thickness) were prepared and stained with hematoxylin-eosin. Each section was photographed under a microscope to observe any pathological changes (400 X magnification).

### Statistical analysis

SPSS was used for statistical evaluation. Data were expressed as the means ± SD (standard deviation). Statistical analyses were performed by one-way ANOVA. Differences at *P* < 0.05 using Duncan’s new multiple-range test were considered statistically significant.

## Additional Information

**How to cite this article**: Liu, M. *et al*. Protective effects on liver, kidney and pancreas of enzymatic- and acidic-hydrolysis of polysaccharides by spent mushroom compost (*Hypsizigus marmoreus*). *Sci. Rep.* **7**, 43212; doi: 10.1038/srep43212 (2017).

**Publisher's note:** Springer Nature remains neutral with regard to jurisdictional claims in published maps and institutional affiliations.

## Supplementary Material

Supplementary Dataset 1
